# Integrating network pharmacology, molecular docking, and experimental validation to investigate the therapeutic effects and potential mechanisms of lycopene against pancreatic ductal adenocarcinoma

**DOI:** 10.3389/fnut.2026.1815515

**Published:** 2026-07-14

**Authors:** Shaoyang Huang, Dandan Gu, Dan Song, Jintao He, Yunpeng Li, Zhengliang Li, Wei Xiong

**Affiliations:** 1 Department of Biochemistry and Molecular Biology, College of Basic Medical Sciences, Dali University, Dali, Yunnan, China; 2 Yunnan Key Laboratory of Screening and Research on Anti-pathogenic Plant Resources from Western Yunnan, College of Pharmacy, Dali University, Dali, Yunnan, China; 3 Department of Cell Biology, College of Life Sciences, Shaanxi Normal University, Xi’an, Shaanxi, China; 4 Department of Gastroenterology, Northeast Yunnan Regional Central Hospital, Zhaotong, Yunnan, China; 5 Department of Urology, The First Affiliated Hospital of Dali University, Dali, Yunnan, China; 6 Department of Radiology, The First Affiliated Hospital of Dali University, Dali, Yunnan, China

**Keywords:** lycopene, molecular docking, network pharmacology, pancreatic ductal adenocarcinoma, PI3K/Akt pathway

## Abstract

**Background:**

Pancreatic ductal adenocarcinoma (PDAC) is an extremely aggressive tumor of the digestive system with a very low five-year survival rate. The limited efficacy and significant toxicity of existing chemotherapy regimens make the development of novel natural therapeutic agents an urgent priority. Lycopene is a natural carotenoid that has been shown to inhibit multiple cancers. However, research specifically targeting PDAC remains relatively scarce.

**Methods:**

This study first employed bibliometric analysis to examine the research landscape and emerging trends in lycopene-related cancer research from 2016 to 2026. Subsequently, network pharmacology methods are applied to screen potential lycopene targets and PDAC-related targets from databases such as CTD, ChEMBL and HERB. Following the identification of overlapping targets, drug-target and protein–protein interaction (PPI) networks are constructed, as well as a disease network. The mechanisms were explored using Gene Ontology (GO) functional enrichment and Kyoto Encyclopedia of Genes and Genomes (KEGG) pathway enrichment analyses. Molecular docking was used to predict the potential interactions between lycopene and representative hub targets, and molecular dynamics simulations were performed for selected high-ranking docking complexes to provide supportive information on complex-level conformational stability. *In vitro* experiments were then conducted to evaluate the predicted anti-PDAC effects and to perform focused validation of apoptosis-related proteins and the PI3K/Akt/P53 signaling axis.

**Results:**

Publications on lycopene research in the field of cancer have shown a sustained upward trend. The focus of this research has gradually shifted from areas such as oxidative stress and antioxidant effects towards anti-cancer mechanisms. A total of 132 overlapping targets for lycopene’s anti-PDAC activity were screened, leading to the identification of 10 core targets, including BCL2, AKT1, and TP53. GO enrichment analysis revealed that these targets are involved in biological processes such as the response to oxidative stress and cellular senescence. Meanwhile, KEGG enrichment analysis identified the PI3K-Akt signaling pathway as a key pathway. Molecular docking results showed that the binding energies of lycopene with core targets such as TP53 and BCL2 were below −4.5 kcal/mol. Molecular dynamics simulations provided supportive evidence for the conformational stability of representative lycopene-target complexes. *In vitro* experiments showed that lycopene inhibited the proliferation and migration of PDAC cells and promoted apoptosis-associated cell death, accompanied by decreased p-PI3K and p-AKT expression and increased P53 expression.

**Conclusion:**

This study systematically combined bibliometrics, network pharmacology, molecular docking, representative molecular dynamics simulations, and focused experimental validation to explore the potential anti-PDAC activity of lycopene. The inflammation-related hub targets identified by network analysis provide additional hypotheses for future experimental investigation. These findings provide preliminary mechanistic evidence for further preclinical investigation of lycopene in PDAC, but its translational application will require optimized formulations, pharmacokinetic validation, and *in vivo* efficacy studies to overcome its limited bioavailability.

## 1 Introduction

With an overall survival rate of less than 10% after 5 years, pancreatic ductal adenocarcinoma (PDAC) is one of the most aggressive cancers of the digestive system. Most individuals are diagnosed with either locally advanced or metastatic disease (1). The approach to treating PDAC is based on the stage of the disease when it is first diagnosed. Systemic therapy plays a pivotal role in the management of PDAC by controlling disseminated disease in advanced stages, as well as in the perioperative setting. Here, neoadjuvant and adjuvant approaches are employed to enhance resectability, delay recurrence, and improve long-term survival outcomes (2, 3). At present, chemotherapy remains the cornerstone of systemic treatment for PDAC. However, conventional regimens, including gemcitabine plus albumin-bound paclitaxel and FOLFIRINOX, only confer modest survival benefits and are frequently associated with substantial toxicity, despite incremental improvements in overall survival (4, 5). Recent advances in molecular biology and precision oncology have catalyzed the development of new chemotherapeutic agents, targeted therapies, and immunotherapeutic approaches, as well as personalized treatment paradigms (6, 7).

Carotenoids represent one of the most extensively distributed groups of pigments in nature, with more than 600 distinct types identified to date (8). In recent years, increasing attention has been paid to the biological functions of carotenoids, particularly their antioxidant, anti-inflammatory, and anticancer activities (9). Lycopene, a red carotenoid pigment originally isolated from tomatoes, has been widely investigated as a natural bioactive compound with potential cancer-preventive properties (10). In addition to its reported associations with reduced risks of prostate and colorectal cancers, epidemiological evidence has suggested a potential inverse association between dietary intake of tomato- and lycopene-rich foods and pancreatic cancer risk, although this relationship requires further validation (11, 12). Importantly, lycopene may be biologically relevant to pancreatic disease because oxidative stress and inflammatory signaling are closely involved in pancreatic tissue injury and PDAC development (13). In cerulein-stimulated pancreatic acinar cells, lycopene was reported to reduce intracellular reactive oxygen species, inhibit NF-κB activation, and suppress IL-6 expression (14). Moreover, in pancreatic cancer PANC-1 cells, lycopene decreased intracellular and mitochondrial ROS levels, inhibited ROS-mediated NF-κB signaling, and promoted apoptosis (15). These findings are particularly relevant to PDAC, in which oncogenic KRAS signaling, oxidative stress, inflammatory pathways, and metabolic reprogramming collectively promote tumor initiation, progression, immune evasion, and therapeutic resistance (16, 17). Therefore, although the role of lycopene in PDAC remains insufficiently explored, its ability to attenuate oxidative stress, suppress inflammation-related signaling, and potentially interfere with tumor-associated metabolic dysregulation provides a mechanistic rationale for investigating its anti-PDAC effects.

To address this knowledge gap, the present study was designed as a stepwise and integrative investigation of the potential anti-PDAC effects of lycopene. First, bibliometric analysis was performed to systematically characterize the research landscape, hotspots, and emerging trends in lycopene-related cancer studies, thereby clarifying the current status of this field and identifying the relative scarcity of PDAC-focused research. Second, network pharmacology was employed to screen the overlapping targets between lycopene and PDAC and to predict the key biological processes and signaling pathways potentially involved in lycopene-mediated anti-PDAC activity. Third, molecular docking was conducted to evaluate the potential interactions between lycopene and representative hub targets identified by network pharmacology. Molecular dynamics simulations were further performed for selected high-ranking docking complexes to provide supportive information on their complex-level conformational stability. Finally, *in vitro* experiments were performed to validate the predicted anti-PDAC effects and biologically relevant signaling pathways, including the regulation of proliferation, migration, apoptosis, EMT, and the PI3K/Akt/P53 signaling pathway. The selection of the PI3K/Akt/P53 axis for experimental validation was mainly based on PPI hub ranking, KEGG enrichment results, and its established biological relevance to PDAC progression.

## 2 Materials and methods

### 2.1 Data analysis based on bibliometrics

We conducted a search of the Web of Science Core Collection (WoSCC) for articles published between January 1, 2016, and January 1, 2026. To minimize the potential influence of subsequent database updates, the full retrieval procedure was completed on a single day using the following search query: TS = (lycopene) AND (tumor OR tumor OR cancer OR neoplasia OR neoplasm OR malignancy OR carcinoma OR adenocarcinoma OR oncology OR lymphoma OR leukemia OR melanoma) AND DOP = (2016-01-01/2026-01-01). The search was restricted to records classified as “Article” according to the Web of Science document type. Articles were included when they were written in English and were relevant to lycopene and cancer research. Other publication types, including reviews, conference abstracts, case reports, letters, editorial materials, and preprints, were excluded. Each retrieved article was carefully assessed to ensure the accuracy of our bibliometric analysis. The inclusion criteria were as follows: (1) relevance to lycopene and tumor research, and (2) publication in English. The exclusion criteria were: (1) Irrelevance to the topic of lycopene and tumor studies; (2) Article types such as reviews, conference abstracts, case reports, letters, and preprints. Full-text versions of the selected articles were then exported. Ultimately, 644 publications focusing on lycopene and tumor research were identified. A visual analysis of annual publication trends, country-specific publication volumes, and trending keywords was conducted using VOSviewer (version 1.6.15) and CiteSpace (version 6.1. R2 Basic Edition).

To further evaluate the disease-specific relevance of lycopene to pancreatic ductal adenocarcinoma, we performed an additional targeted search using PDAC-related terms. The secondary search strategy was as follows: TS = (“lycopene”) AND TS = (“pancreatic cancer” OR “pancreatic carcinoma” OR “pancreatic neoplasm” OR “cancer of pancreas” OR “carcinoma of pancreas” OR “neoplasm of pancreas” OR “pancreatic ductal adenocarcinoma” OR PDAC). The retrieved records were manually screened to identify studies directly related to lycopene and pancreatic cancer/PDAC. Because only a limited number of eligible PDAC-specific publications were identified, this subset was summarized descriptively rather than subjected to bibliometric network visualization, keyword clustering, or burst detection analysis. The identified PDAC-related studies are summarized in Supplementary Table S1.

### 2.2 Drugs and reagents

Lycopene (purity ≥98.0%, product no. S3943) was obtained from Selleck Chemicals. Lycopene was dissolved in dimethyl sulfoxide (DMSO) to prepare a 40 mM stock solution and diluted with culture medium immediately before use. In all *in vitro* experiments, the final concentration of DMSO was kept constant at 0.1% v/v in both the vehicle control and lycopene-treated groups. The vehicle control group received the same concentration of DMSO without lycopene. The antibodies utilized for Western blot analysis in this study were as follows: GAPDH (Proteintech, Cat. No. 60004-1-Ig, diluted at 1:1000); E-cadherin (Proteintech, Cat. No. 20874-1-AP, diluted at 1:2000); Vimentin (Proteintech, Cat. No. 10366-1-AP, diluted at 1:2000); Cleaved-caspase-3 (Abcam, Cat. No. ab2302, diluted at 1:1000); BCL2 (Abcam, Cat. No. EPR17509, diluted at 1:1000); Bax (Abcam, Cat. No. ab32503, diluted at 1:1000); p-PI3K (Affinity, Cat. No. AF3242, diluted at 1:1000); PI3K (Affinity, Cat. No. AF6241, diluted at 1:1000); p-AKT (CST, Cat. No. 4060S, diluted at 1:1000); AKT (Abcam, Cat. No. ab8805, diluted at 1:1000); P53 (Proteintech, Cat. No. 10442-1-AP, diluted at 1:1000); Goat anti-rabbit IgG secondary antibody-HRP-labeled (Abcam, Cat. No. ab6721, diluted at 1:5000).

### 2.3 Target screening for lycopene

The chemical information of lycopene was obtained from PubChem^1^ using “lycopene” and its PubChem CID as search terms. Lycopene-related targets were collected from the Comparative Toxicogenomics Database (CTD) platform^2^, ChEMBL database^3^, and HERB database^4^. To improve reproducibility, the following screening criteria were applied: for CTD, only curated chemical-gene/protein interactions directly associated with lycopene and supported by at least one reference were retained; for ChEMBL, records were retained when the assay confidence score was ≥7, the activity type was IC_50_, EC_50_, Ki, or Kd, and the activity value was ≤10 μM, corresponding to pChEMBL ≥5; for HERB, targets were retained when they had explicit compound-target relationships, confidence score ≥0.7, and complete target annotation. Across all databases, only *Homo sapiens* targets with official gene symbols were included. Non-protein targets, duplicate records, and entries with incomplete or ambiguous annotations were excluded. All retained targets were standardized using UniProt before subsequent analysis.

### 2.4 Identification of PDAC targets

PDAC-related targets were collected from The Cancer Genome Atlas database (TCGA^5^), the Gene Expression Omnibus database (GEO^6^), and the GeneCards database^7^ on accessed in February 2026. For TCGA and the GEO dataset GSE62452, differentially expressed genes between PDAC and non-tumor pancreatic tissues were screened using the criteria of *p* < 0.05 and |*logFC*| > 2. For GeneCards, “pancreatic ductal adenocarcinoma” was used as the search term, and genes with a relevance score ≥10 were retained as PDAC-associated targets. All retrieved genes were standardized to official gene symbols using the UniProt database. Genes with incomplete or ambiguous annotations were excluded. Finally, the standardized PDAC-related genes obtained from TCGA, GSE62452, and GeneCards were merged, and duplicate genes were removed to generate the final PDAC-related target set for subsequent analysis.

### 2.5 Construction of drug-target-disease networks

The “VennDiagram” package in R software was employed to identify the intersection of target genes associated with lycopene’s anti-PDAC activity. The Cytoscape software was then used to construct regulatory networks of drugs, targets, and diseases, facilitating a comprehensive and logical interpretation of the interrelationships between compounds and their respective targets.

### 2.6 Construction of the protein–protein interaction (PPI) network for common targets of lycopene and PDAC

The overlapping targets of lycopene and PDAC were imported into the STRING database^8^ to construct the PPI network. The organism was restricted to *Homo sapiens*, and the minimum required interaction score was set to 0.7, which represents a high-confidence threshold in STRING. Disconnected nodes were hidden from the network. The resulting TSV file was downloaded and imported into Cytoscape 3.9.0 for visualization and topological analysis. To identify hub genes, the CytoHubba plugin was used. The top 10 hub genes were first screened using the Maximum Clique Centrality algorithm. To further evaluate the robustness of hub-gene selection, additional topological algorithms, including Degree, Betweenness, Closeness, Maximum Neighborhood Component, and Density of Maximum Neighborhood Component, were also applied. Genes repeatedly ranked among the top candidates by multiple algorithms were considered robust hub genes and were selected for subsequent molecular docking and experimental validation.

### 2.7 GO enrichment analysis and KEGG pathway enrichment analysis

To investigate the biological functions and signaling pathways associated with the overlapping targets of lycopene and PDAC, GO and KEGG enrichment analyses were performed using the R package clusterProfiler. All target genes were converted to official gene symbols and Entrez IDs before enrichment analysis. The background gene set was defined as all human genes with valid Entrez IDs and GO or KEGG annotations. The Benjamini-Hochberg method was used for multiple-testing correction. Enriched GO terms and KEGG pathways were considered statistically significant when the adjusted *p* value (*p.adjust*) < 0.05 and *q*-value < 0.05. The number of enriched genes, gene ratio, background ratio, raw *p* value, *p.adjust*, *q*-value, and enriched gene symbols were recorded for each term or pathway. The top 10 GO terms in Biological Process (BP), Cellular Component (CC), and Molecular Function (MF) categories and the top 20 KEGG pathways were visualized using the R package ggplot2. The complete GO and KEGG enrichment results were provided in Supplementary Tables S2, S3, respectively.

### 2.8 Molecular docking

The three-dimensional structures of the core target proteins were obtained from the Protein Data Bank (PDB). The three-dimensional structure of lycopene was downloaded from PubChem and energy-minimized before docking. Protein structures were prepared using AutoDock Tools by removing water molecules and original ligands, adding polar hydrogen atoms, and assigning Gasteiger charges. The prepared proteins and ligand were saved in PDBQT format. Molecular docking was performed using AutoDock 4. For target proteins with co-crystallized ligands, the docking region was defined according to the position of the original ligand. For proteins without co-crystallized ligands, the docking region was determined based on reported functional domains or predicted binding pockets. The docking grid was adjusted to cover the corresponding binding pocket of each target protein. The Lamarckian genetic algorithm was used for conformational searching. The docking pose with the lowest binding energy was selected as the optimal docking conformation. Binding affinity was evaluated based on the calculated binding energy, with a more negative binding energy indicating stronger predicted binding stability. The docking conformations and ligand-residue interactions were visualized using PyMOL.

### 2.9 Molecular dynamics

To further explore the dynamic behavior of representative protein-lycopene complexes obtained from molecular docking, molecular dynamics simulations were performed using GROMACS 2022. Based on the molecular docking scores, BCL2-lycopene and TP53-lycopene, which showed the most favorable predicted docking energies among the selected hub targets, were chosen for 100 ns molecular dynamics simulations. AKT1 was not included in the MD simulation because its docking score was not among the top-ranked complexes; however, AKT1 was retained for subsequent experimental validation based on PPI hub ranking, KEGG pathway enrichment of the PI3K-Akt signaling pathway, and its biological relevance to PDAC progression.

The CHARMM36 force field was used for protein parameterization, and lycopene was parameterized using CHARMM General Force Field-compatible ligand parameters. Each protein-ligand complex was placed in a dodecahedral simulation box and solvated with the TIP3P water model. Counterions were added to neutralize the total charge of the system. Energy minimization was first performed using the steepest descent algorithm, followed by equilibration under NVT and NPT ensembles. The temperature was maintained at 310 K, and the pressure was maintained at 1 atm. After equilibration, a 100 ns production molecular dynamics simulation was performed for each selected complex.

The dynamic behavior of the simulated complexes was evaluated using protein backbone root mean square deviation, root mean square fluctuation, radius of gyration, solvent-accessible surface area, and relative Gibbs free energy landscape analyses. Because apo-protein simulations and ligand-specific stability analyses were not included in the present study, RMSF results were interpreted only as residue-level fluctuation profiles of the simulated complexes and were not used to directly attribute flexibility changes to lycopene binding. The relative Gibbs free energy landscape was calculated based on the conformational distribution of the simulated complexes. The minimum free energy state was set as the reference state; therefore, the values shown in the free energy landscape represent relative free energy differences rather than absolute binding free energies.

### 2.10 Cell culture

The American Type Culture Collection (ATCC) in Manassas, USA, provided the human PDAC cell lines (SW1990, PANC-1) and normal pancreatic duct epithelial cells (HPNE, HPDE6-C7). DMEM medium (Invitrogen, Carlsbad, CA, USA) with 1% penicillin–streptomycin and 10% fetal bovine serum (FBS) were utilized to culture the cells. Every cell line was kept at 37 °C in a humidified incubator with 5% CO₂. The medium was changed every 2–3 days, and trypsin was added for digestion and passage when the cells were 80% confluent. Short tandem repeat (STR) profiling was used to check all the cell lines, and mycoplasma status was checked often using the Luciferase Mycoplasma Detection Kit (TransGen Biotech., Beijing, China).

### 2.11 Cell proliferation assay

After being seeded into 96-well plates, the cells were exposed to different concentrations of lycopene or the corresponding DMSO vehicle control for either 48 or 72 h. The final concentration of DMSO was kept constant at 0.1% v/v in all groups. Cell viability was assessed using the Cell Counting Kit-8 (Cat. No. CK04, Dojindo, Japan), and the absorbance at 450 nm was measured using a microplate reader (Pharmacia Biotech). Three replicate wells were made up for each group, and the experiment was carried out three times on its own. The half-maximal inhibitory concentration (IC₅₀) was determined using GraphPad Prism 7.0. Dose–response curves were generated based on average absorbance values, and the IC₅₀ was calculated using a four-parameter logistic model via nonlinear regression.

### 2.12 Cell colony formation

PDAC cells were seeded into six-well plates at a density of 1,000 cells per well. After cell attachment, the cells were treated with DMSO vehicle control or 20 or 40 μM lycopene and cultured for 14 days to allow colony formation. The final DMSO concentration was kept constant at 0.1% v/v in all groups. The culture medium containing the corresponding concentration of lycopene was replaced every 3 days. At the end of the incubation period, colonies were fixed with 4% paraformaldehyde, stained with crystal violet for 15 min, air-dried, and photographed. Colonies containing more than 50 cells were counted for statistical analysis. All tests were performed in triplicate.

### 2.13 Wound healing assay

PDAC cells were cultured in six-well plates until a confluent monolayer was formed. A linear scratch wound was created on each culture plate using the tip of a 10 μL pipette. After removing unattached cells by PBS washing, serum-free medium containing DMSO vehicle control or 20 or 40 μM lycopene was added. The final DMSO concentration was kept constant at 0.1% v/v in all groups. After 48 h of cell culture, the wound area was observed under an inverted microscope, and images were captured for analysis. All tests were performed in triplicate.

### 2.14 Transwell assay

The lower chamber received 500 μL of medium containing 20% FBS, whereas the top chamber received 200 μL of serum-free medium containing 4 × 10^4^ cells. After the cells had adhered, serum-free medium containing DMSO vehicle control or 20 or 40 μM lycopene was added to the upper chamber. The final DMSO concentration was kept constant at 0.1% v/v in all groups. Following incubation, the cells were fixed and stained with crystal violet. The number of migrating cells in each area of the slide was then counted under a microscope. All tests were performed in triplicate.

### 2.15 Flow cytometry

After being incubated with DMSO vehicle control or lycopene for 24 h, PDAC cells were trypsinized and centrifuged. The final DMSO concentration was kept constant at 0.1% v/v in all groups. Following three PBS buffer washes, Buffer solution was used to resuspend the cells. After that, the reconstituted cells were co-incubated for 15 to 20 min with 5 μL of Annexin V-fluorescein isothiocyanate (Annexin V-FITC). For the analysis of apoptosis, 5 μL of 7-Aminoactinomycin D (7-AAD) was added to the mixture, which was then incubated for a further 5 min. BD FACSVerse™ flow cytometry was used to measure apoptosis in PDAC cells. All tests were performed in triplicate.

### 2.16 Western blotting analysis

Protease and phosphatase inhibitors were added to the RIPA lysis buffer (Cat. No. P0013B, Beyotime, China) and used to lyse cells on ice for 15 min. The protein concentration in the cell lysate was then determined using the bicinchoninic acid (BCA) Protein Assay Kit (Cat. No. P0012, Beyotime, China). Total protein lysates from various samples were separated by 8–12% sodium dodecyl sulfate-polyacrylamide gel electrophoresis (SDS-PAGE) and transferred to a polyvinylidene fluoride (PVDF) membrane using an electrophoretic transfer apparatus (100 V, 4 °C, 1 h). After blocking with blocking buffer, the membrane was incubated with the primary antibody overnight at 4 °C. The following day, the membrane was washed with buffered saline with tween-20 (TBST) and incubated with an horseradish peroxidase (HRP)-labeled secondary antibody. Protein bands were visualized using enhanced chemiluminescence (ECL) and exposed to radiographic film. The relative expression level of the protein is quantified by calculating the ratio of the accumulated optical density (AOD) of the target band to that of the internal reference band. Semi-quantitative analysis was performed with ImageJ software. Three independent replicates were used in every experiment.

### 2.17 Statistical analysis

GraphPad Prism 7.0 and SPSS 20.0 were used for statistical analysis. Each experiment was conducted at least three times. The mean ± standard deviation (mean ± SD) was used to display the data. *p*-values below 0.05 were considered statistically significant. The chi-square (*χ^2^*) test, Student’s *t*-test, and one-way analysis of variance (ANOVA) were used to compare group means.

## 3 Results

### 3.1 Bibliometric analysis of lycopene in cancer research: publication trends, hotspots, and frontiers from 2016 to 2026

**Figure 1:**
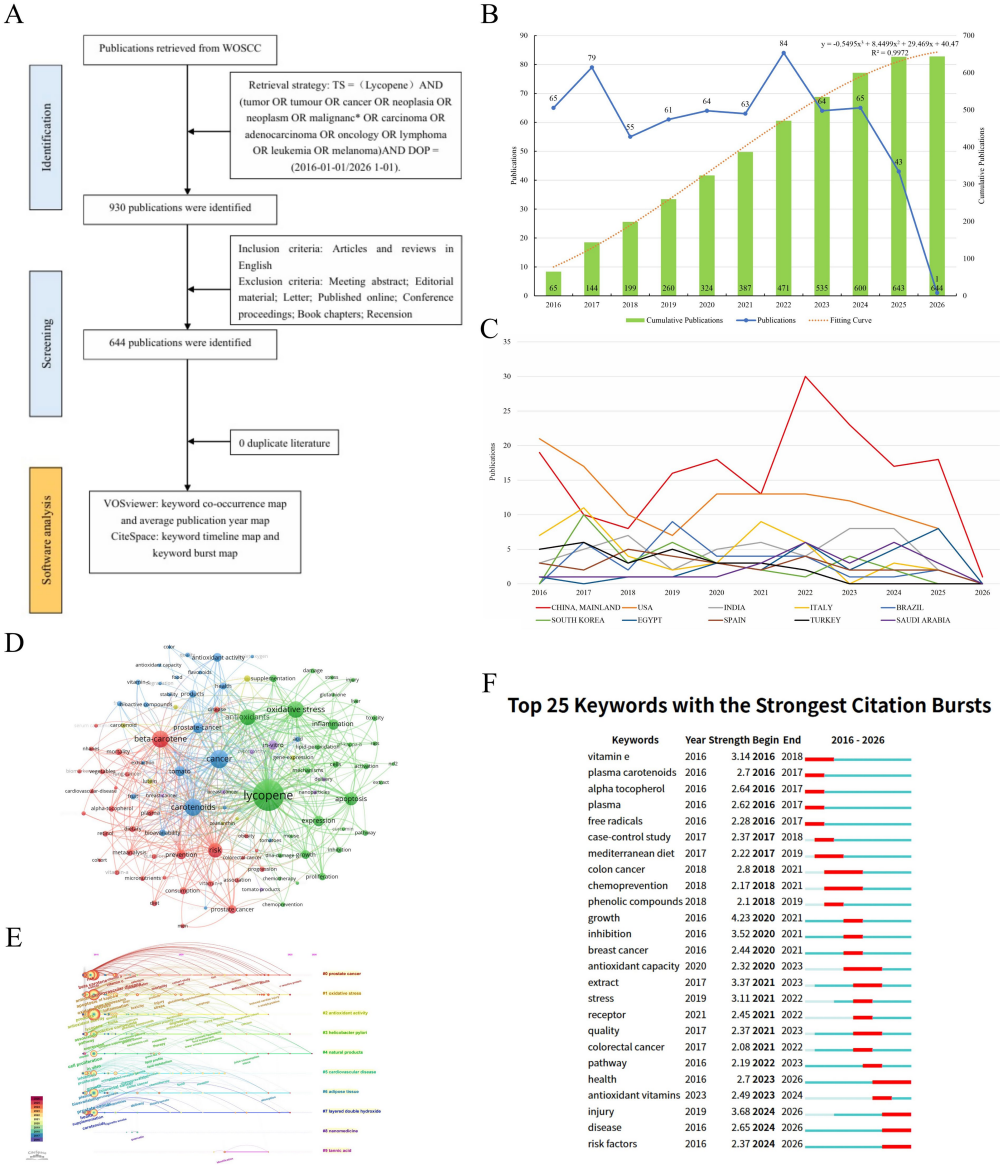
Research advances and hot trends in lycopene within the field of oncology. **(A)** Literature screening flowchart. **(B)** Annual publication volume and cumulative publication trend chart. **(C)** Annual trend chart of publication volume by major countries/regions. **(D)** Research keyword co-occurrence network map. **(E)** Research keyword timeline map. **(F)** Top 25 keywords with highest citation intensity: emergent analysis chart.

Given the current lack of systematic reviews on the status and progress of lycopene in cancer research, we employed bibliometric analysis, a quantitative method of analyzing academic publications, to evaluate the current state and emerging trends of lycopene in cancer studies. The screening process in its entirety is illustrated in Figure 1A. The annual publication trend shown in Figure 1B and the statistical table of publication volumes by country (Table 1) reveal a consistent increase in lycopene-related cancer research publications from 2016 to 2025. The fitted curve indicates a significant growth trajectory, with accelerated growth observed from 2021 to 2025, peaking at 643 publications in 2025. The country/region publication distribution (Figure 1C) shows that China, the United States, Italy, Spain, and Turkey are the leading contributors to publications in this field. The keyword co-occurrence network (Figure 1D) and the high-frequency keyword list (Table 2) demonstrate that “lycopene” is a central keyword, forming a tightly connected network with terms such as “oxidative stress,” “cancer,” “antioxidant,” and “beta-carotene,” underscoring its pivotal role in research on cancer-related oxidative stress regulation. The keyword timeline evolution (Figure 1E) clearly illustrates the shifting research priorities: from 2016 to 2018, the focus was on the relationship between antioxidants like vitamin E and carotenoids and cancer; from 2019 to 2021, attention shifted to specific cancer types and intervention strategies, including chemoprevention, breast cancer, and colorectal cancer; from 2022 to 2026, research concentrated on antioxidant vitamins, damage, disease, and risk factors. These evolving keywords reflect a growing emphasis on lycopene’s role in disease prevention and cancer risk regulation. The keyword emergence intensity analysis in Figure 1F further supports this trend. Early emerging terms were primarily focused on antioxidant-related basic research, with a shift in the mid-period toward clinical and population studies, and more recently, a focus on anti-cancer mechanisms, key pathways, and health effects. The overall upward trend in emergence intensity highlights the field’s increasingly in-depth exploration of lycopene. A secondary targeted search was performed to evaluate the PDAC-specific evidence for lycopene. Only a limited number of eligible studies directly related to lycopene and pancreatic cancer/PDAC were identified after manual screening. Therefore, this subset was summarized descriptively in Supplementary Table S1 rather than being subjected to bibliometric visualization, indicating that direct research on lycopene and PDAC remains scarce.

**Table 1:** Statistics of top countries in terms of number of publications (top 10).

Rank	Country	Publications (644, %)
1	China	173 (26.86%)
2	USA	124 (19.25%)
3	India	50 (7.76%)
4	Italy	47 (7.30%)
5	Brazil	33 (5.12%)
6	South Korea	31 (4.81%)
7	Egypt	29 (4.50%)
8	Spain	29 (4.50%)
9	Turkey	27 (4.19%)
10	Saudi Arabia	26 (4.04%)

**Table 2:** The top 20 high-frequency keywords.

Rank	Label	Occurrences
1	Lycopene	405
2	Cancer	162
3	Carotenoids	148
4	Beta-carotene	141
5	Oxidative stress	138
6	Antioxidants	128
7	Risk	108
8	Apoptosis	90
9	Expression	66
10	Inflammation	66
11	Prostate-cancer	63
12	Tomato	60
13	*In vitro*	56
14	Prevention	52
15	Growth	48
16	Antioxidant activity	47
17	Prostate cancer	44
18	Supplementation	36
19	Products	35
20	Proliferation	35

### 3.2 Drug target sets and PDAC-related targets

**Figure 2:**
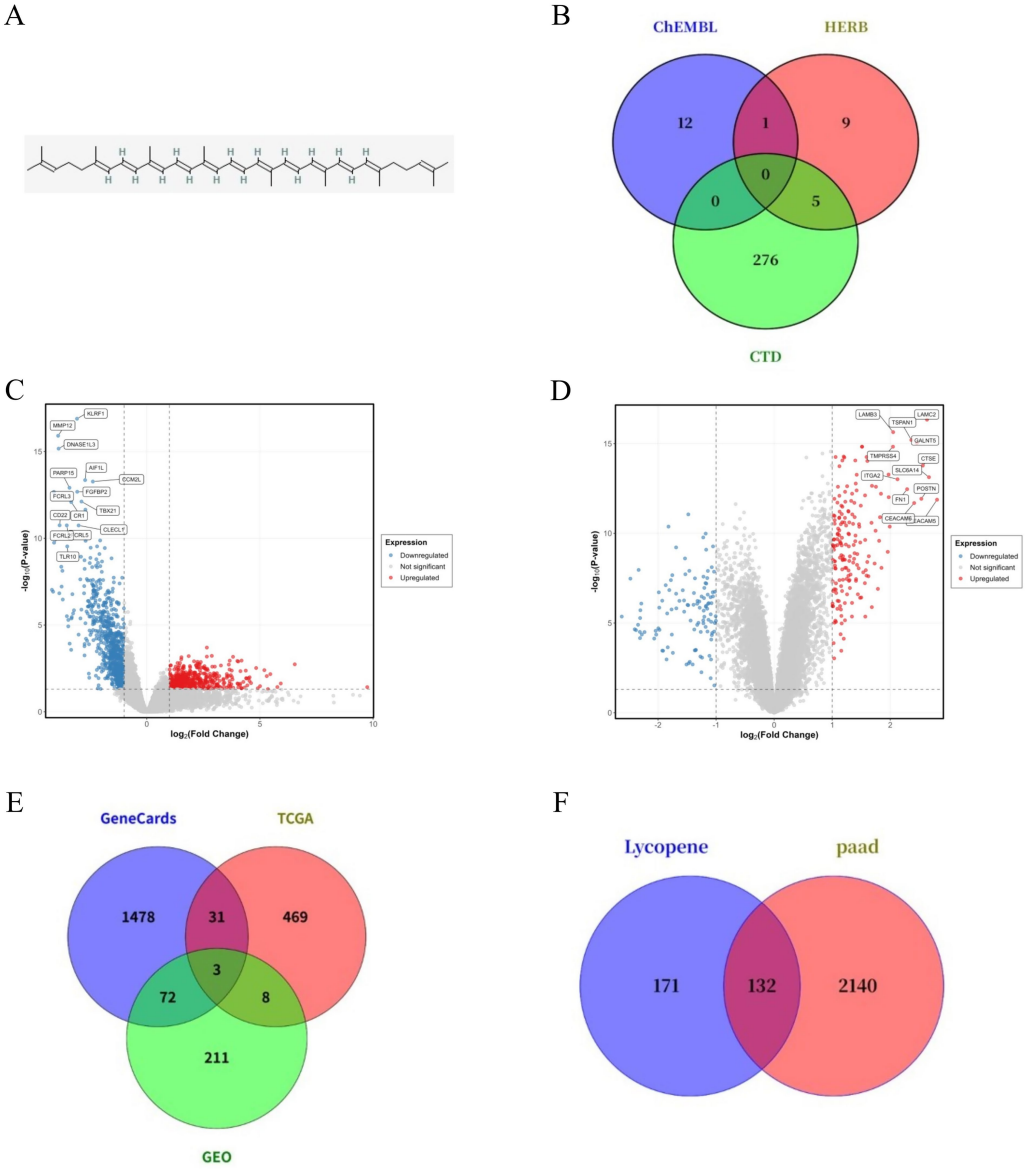
Screening of potential targets for lycopene in combating PDAC and bioinformatics analysis. **(A)** Chemical structure formula of lycopene. **(B)** Venn diagram showing the intersection of lycopene-related targets across different databases (ChEMBL, HERB, CTD). **(C)** Volcano plot of differentially expressed genes between PDAC and adjacent normal tissues in the TCGA dataset. **(D)** Volcano plot of differentially expressed genes between PDAC tissue and adjacent non-cancerous tissue in GEO. **(E)** Venn diagram showing the intersection of PDAC differentially expressed genes across GeneCards, TCGA, and GEO databases. **(F)** Venn diagram showing the intersection of lycopene targets and PDAC differentially expressed genes.

Lycopene is a natural carotenoid compound, and its chemical structure is shown in Figure 2A. To identify potential therapeutic targets of lycopene against PDAC, two independent target sets were first constructed. The first set consisted of lycopene-related targets obtained from the ChEMBL, HERB, and CTD databases. After screening, standardization, and duplicate removal, 303 potential lycopene-associated target genes were identified (Figure 2B). The second set consisted of PDAC-related targets obtained from TCGA, the GEO dataset GSE62452, and the GeneCards database. Differentially expressed genes from TCGA and GSE62452 were screened and integrated with PDAC-associated genes from GeneCards, resulting in 2,272 potential PDAC-related targets (Figures 2C–E). The lycopene-related target set and the PDAC-related target set were then intersected to identify candidate targets that were both associated with lycopene and involved in PDAC. A total of 132 overlapping genes were obtained (Figure 2F). These overlapping genes were considered potential targets through which lycopene may exert therapeutic effects against PDAC and were used for subsequent PPI network construction, functional enrichment analysis, molecular docking, and experimental validation.

### 3.3 Construction of compound-target-disease (CTD) networks and PPI networks

**Figure 3:**
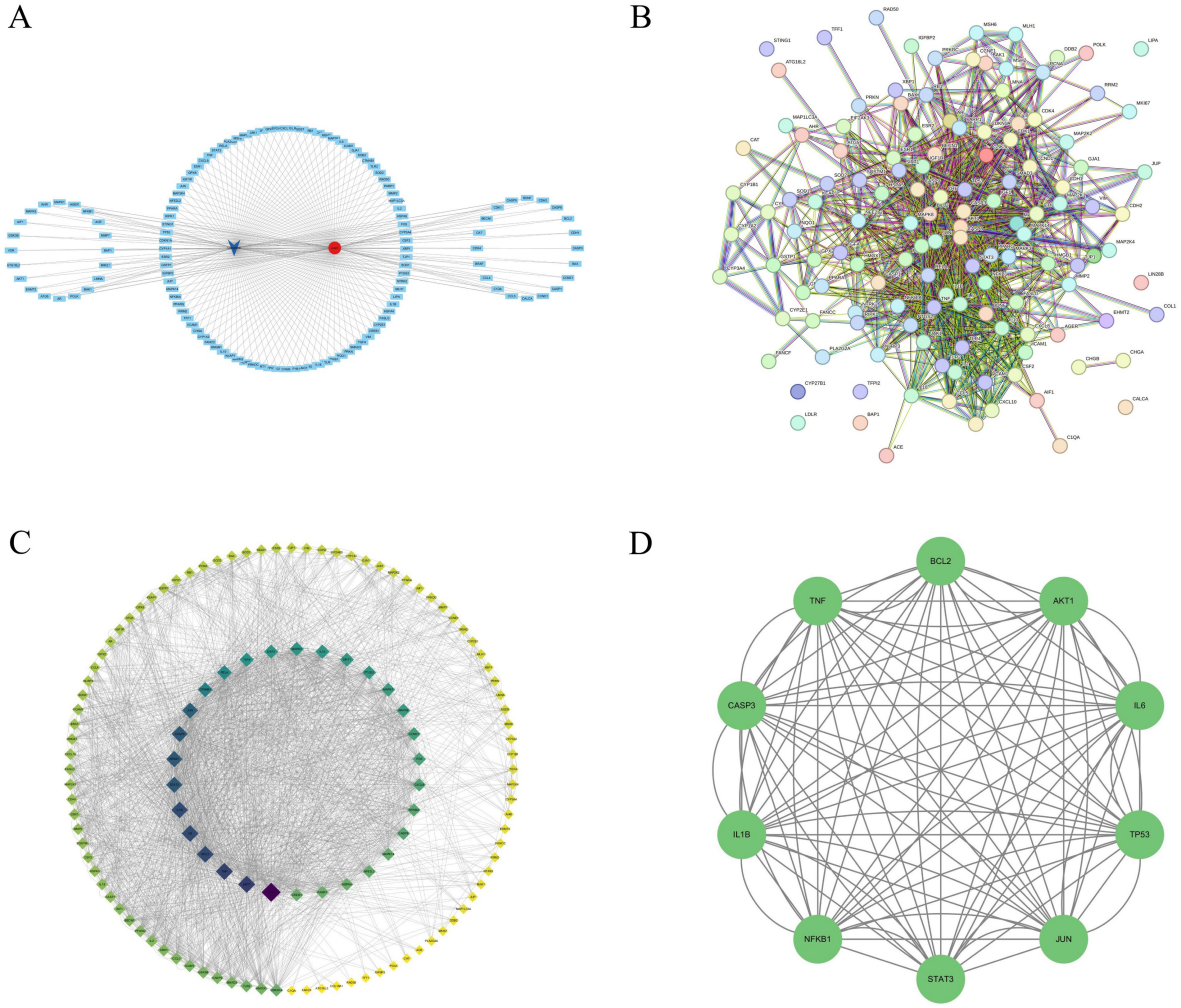
Core target protein interaction network and key gene screening of lycopene in PDAC prevention. **(A)** Preliminary construction of the lycopene-PDAC target interaction network. **(B)** The PPI network of lycopene targeting PDAC. **(C)** Core target network based on degree-based screening. **(D)** Top 10 key core gene interaction networks of lycopene in combating PDAC.

A CTD network linking substances, targets, and illnesses was built using Cytoscape software to clarify the pharmacological mechanism of lycopene in the management of PDAC (Figure 3A). Nodes in this network represent the 132 overlapping genes (blue), lycopene (dark blue), and PDAC (red). The results suggest that lycopene exerts anticancer effects on PDAC cells through multiple targets. Additionally, a PPI network was constructed using data from the STRING database to investigate the potential mechanism underlying lycopene’s anti-PDAC activity (Figure 3B). Cytoscape 3.9.0 software was further utilized to construct and analyze the PPI network of intersecting targets, offering insights into the potential mechanism of lycopene therapy for PDAC. The degree values of all nodes were calculated and visualized using a circular layout, with larger node areas representing higher degree values. In Figure 3C, in the inner layer, the top 30 target nodes with degree values greater than 50 are shown in different colors. The top ten hub genes, including BCL2, AKT1, IL6, TP53, JUN, STAT3, NFKB1, IL1B, CASP3, and TNF, were identified based on the PPI network analysis and are highlighted in (Figure 3D). These hub genes were considered potential core targets involved in the anti-PDAC effects of lycopene. It should be noted that these hub genes were identified through network-based prediction and should be interpreted as candidate targets rather than experimentally validated mediators at this stage. Among them, six representative targets, TP53, AKT1, TNF, BCL2, IL6, and IL1B, were selected for subsequent molecular docking analysis based on their network importance, biological relevance to PDAC progression, and availability of suitable three-dimensional protein structures.

### 3.4 GO enrichment and KEGG pathway analysis

**Figure 4:**
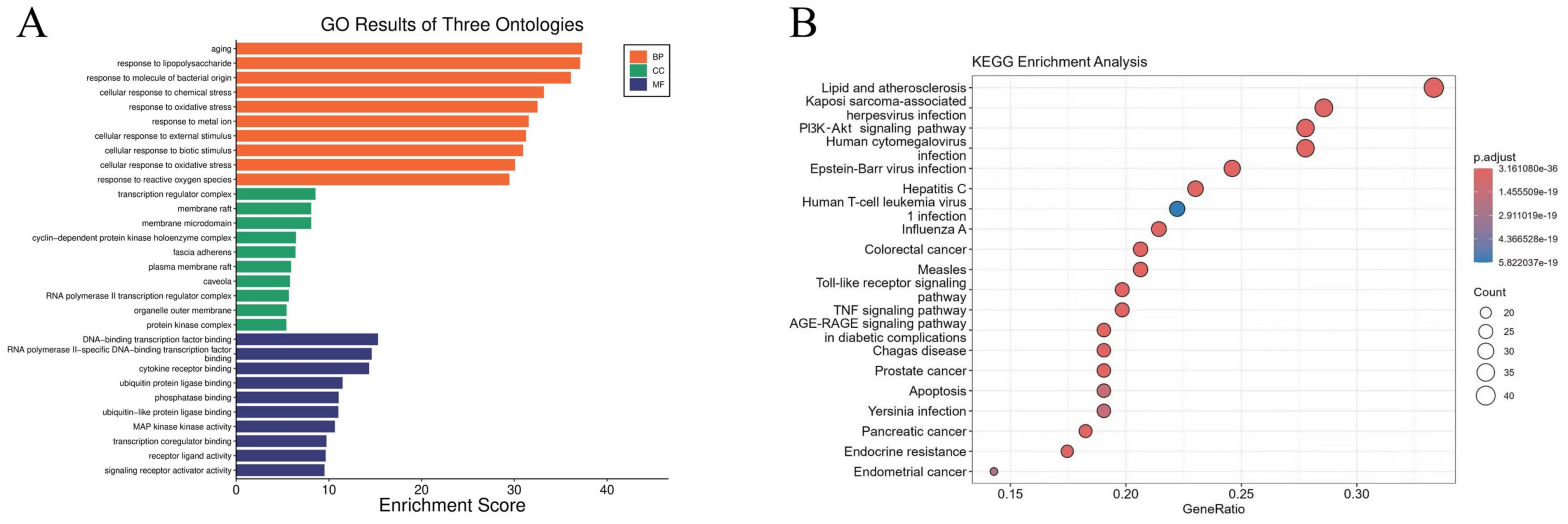
GO functional enrichment and KEGG pathway enrichment analysis of core targets for lycopene’s anti-PDAC effects. **(A)** Bar chart of GO functional enrichment analysis for core targets of lycopene in PDAC prevention. **(B)** KEGG pathway enrichment analysis bubble chart for lycopene’s core target in PDAC prevention.

This study examined the impact of lycopene on biological processes and signaling pathways linked to PDAC development using GO and KEGG enrichment analysis on common genes. GO annotation analysis encompasses three major categories: BP, CC, and MF. Bar charts illustrate the significantly enriched entries across these categories. Figure 4A shows that, at the MF level, genes were predominantly enriched in DNA-binding transcription factor binding and MAP kinase kinase activity. At the BP level, genes were significantly enriched in processes related to cellular stress and immunity, such as the response to oxidative stress and aging. Figure 4B presents the results of a KEGG enrichment analysis, revealing that the genes are significantly enriched in multiple pathways associated with tumor progression. These pathways include the PI3K-Akt signaling pathway, the Toll-like receptor signaling pathway, as well as pathways linked to prostate and colorectal cancer. In conclusion, the findings of the enrichment analysis show that the core targets are closely linked to several signaling pathways, with the PI3K/AKT pathway demonstrating the strongest enrichment.

### 3.5 Molecular docking and molecular dynamics simulation

**Figure 5:**
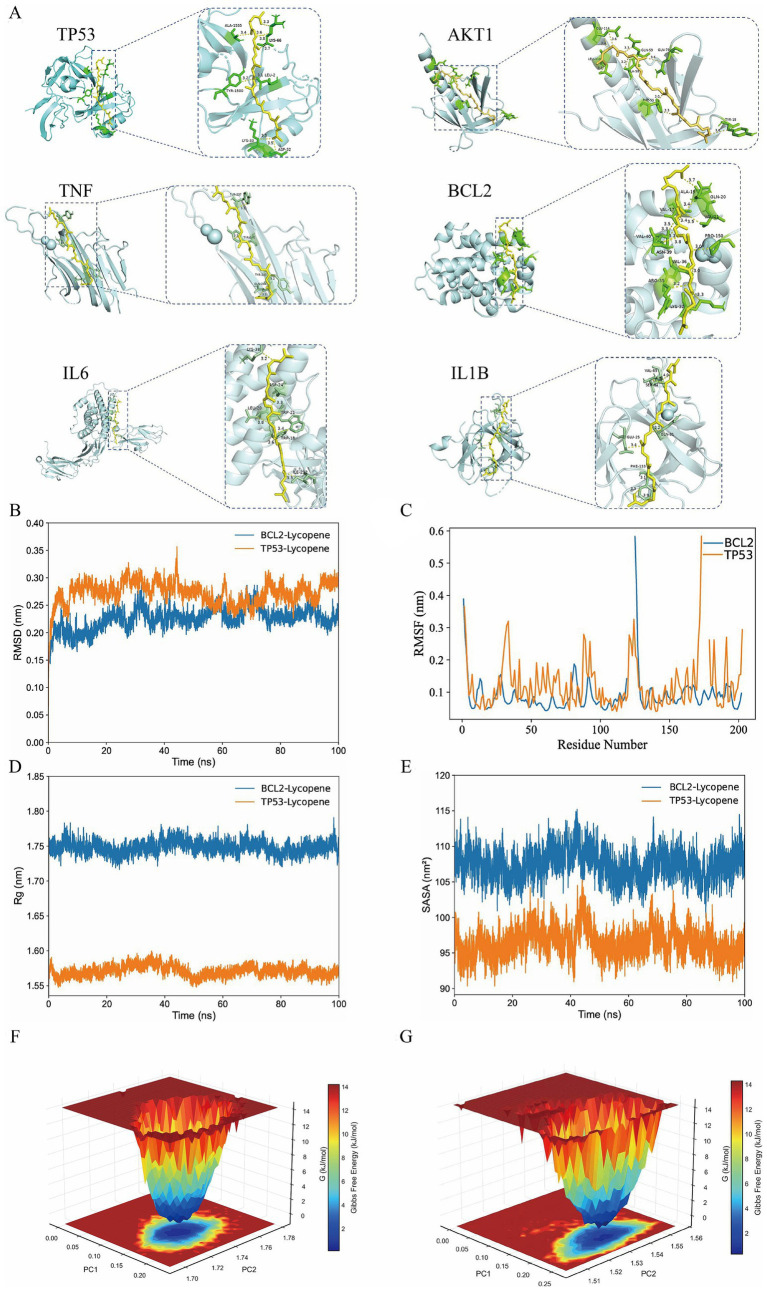
Molecular docking and molecular dynamics simulation of lycopene with key target sites in PDAC. **(A)** Molecular docking conformations of lycopene with key target proteins, including TP53, AKT1, TNF, BCL2, IL6, and IL1B. **(B)** RMSD curves of the BCL2-lycopene and TP53-lycopene complexes during 100 ns molecular dynamics simulations. **(C)** RMSF curves of BCL2 and TP53 proteins during the simulations. Because apo-protein simulations were not included, RMSF profiles were used only to describe residue-level fluctuations within the simulated complexes. **(D)** Rg curves of the BCL2-lycopene and TP53-lycopene complexes during 100 ns simulations. **(E)** SASA curves of the BCL2-lycopene and TP53-lycopene complexes during 100 ns simulations.**(F)** Relative Gibbs free energy landscape of the BCL2-lycopene complex. **(G)** Relative Gibbs free energy landscape of the TP53-lycopene complex. The free energy landscape was used to describe the relative conformational distribution of the simulated complexes. The minimum free energy state was set as the reference state, and the plotted values represent relative Gibbs free energy differences rather than absolute binding free energies.

Molecular docking was performed to evaluate the potential interactions between lycopene and six representative hub targets, including TP53, AKT1, TNF, BCL2, IL6, and IL1B. These proteins were selected from the top 10 hub genes based on their network ranking, biological relevance to PDAC progression, and availability of suitable three-dimensional structures. The optimal docking conformations were visualized using PyMOL software (Figure 5A). The predicted docking energies of lycopene with TP53, AKT1, TNF, BCL2, IL6, and IL1B were −7.68, −5.93, −5.94, −7.85, −5.80, and −7.66 kcal/mol, respectively (Table 3). These results suggested favorable predicted interactions between lycopene and these representative hub targets. To further explore the dynamic behavior of representative lycopene-target complexes, 100 ns molecular dynamics simulations were performed for BCL2-lycopene and TP53-lycopene, which showed the most favorable docking scores among the selected targets. AKT1 was not included in the MD simulation because its docking score was not among the top-ranked complexes. However, AKT1 was retained for subsequent experimental validation because it was identified as a hub target in the PPI network and because KEGG enrichment analysis highlighted the PI3K-Akt signaling pathway as a major pathway potentially involved in lycopene-mediated anti-PDAC effects.

**Table 3:** Molecular docking energy results of lycopene with selected target proteins.

Protein-ligand complex	△G/(Kcal/mol)	Eintermol/(Kcal/mol)	Evhd/(Kcal/mol)	Eelec/(Kcal/mol)
TP53-lycopene	−7.68	−12.43	−12.39	−0.05
AKT1-lycopene	−5.93	−10.71	−10.7	−0.01
TNF-lycopene	−5.94	−10.71	−10.74	0.02
BCL2-lycopene	−7.85	−12.62	−12.6	−0.02
IL6-lycopene	−5.8	−10.57	−10.57	−0.01
IL1B-lycopene	−7.66	−12.45	−12.46	0.01

The RMSD curves of the BCL2-lycopene and TP53-lycopene complexes showed relatively stable fluctuation patterns during the 100 ns simulations, suggesting that the overall conformations of these simulated complexes did not undergo major structural disruption (Figure 5B). RMSF analysis was used to describe residue-level flexibility within the simulated complexes (Figure 5C). Because apo-protein simulations were not included in the present study, the RMSF results were interpreted cautiously and were not used to directly attribute changes in protein flexibility to lycopene binding. The Rg curves remained relatively stable during the simulations, indicating that the overall compactness of the complexes was largely maintained (Figure 5D). SASA analysis also showed relatively stable solvent exposure profiles during the simulations (Figure 5E). Figures 5F,G show the relative Gibbs free energy landscapes of the BCL2-lycopene and TP53-lycopene complexes, respectively. The free energy landscape analysis was used to describe the relative conformational distribution of the simulated complexes rather than absolute binding free energy. Overall, these MD results provide supportive computational evidence for the conformational stability of representative lycopene-target complexes, but they do not prove direct biochemical binding between lycopene and the target proteins.

### 3.6 Lycopene inhibits proliferation of PDAC cells

**Figure 6:**
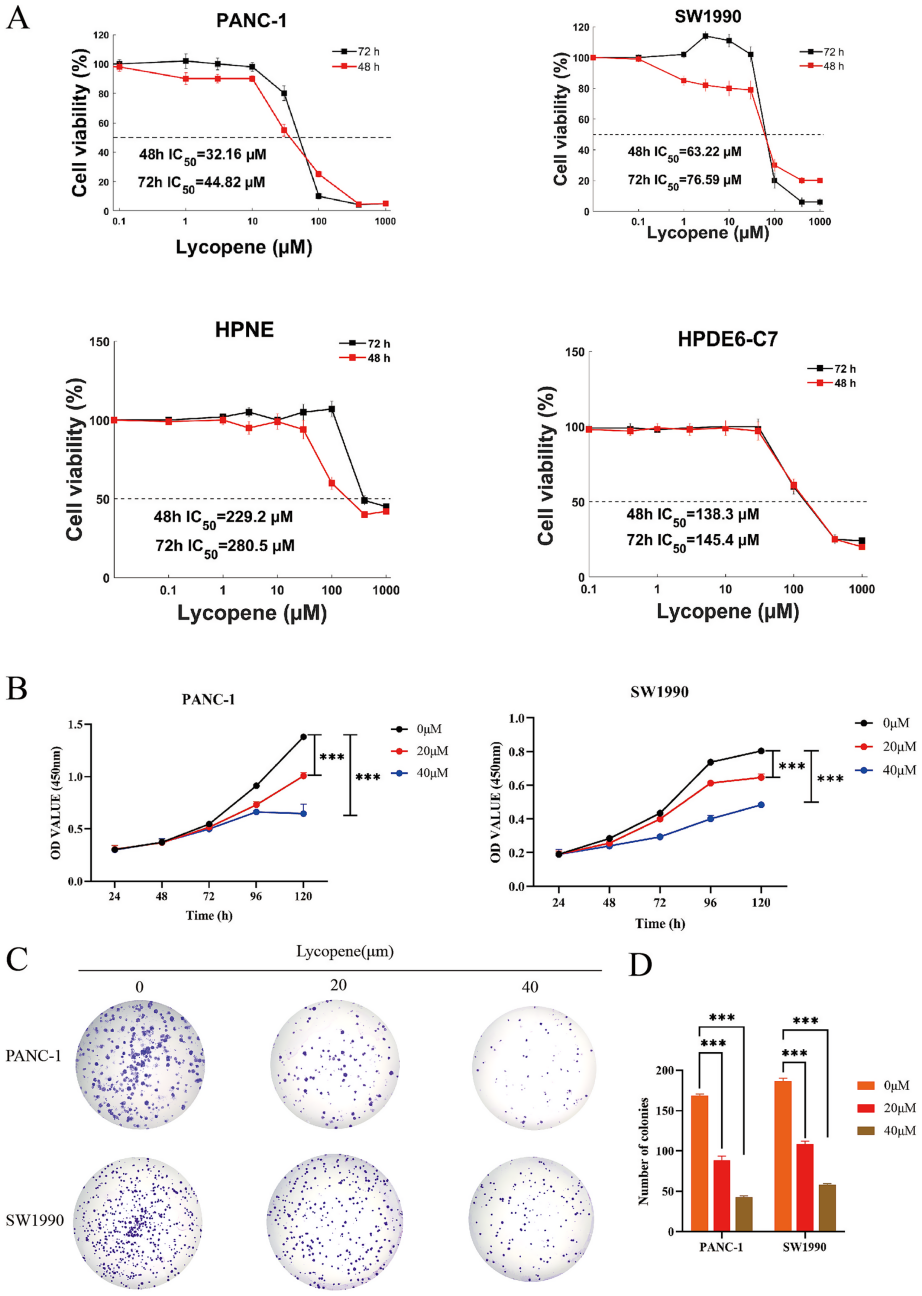
Lycopene inhibits the proliferation of PDAC cells. **(A)** CCK-8 assay for detecting the viability of PANC-1, SW1990, HPNE, and HPDE6-C7 cells after 48 and 72 h of lycopene treatment (*n* = 3). **(B)**Growth curves of PANC-1 and SW1990 cells over 120 h following treatment with different concentrations of lycopene. **(C)** Results of the colony formation assay after 14 days of co-culture with tomato lycopene following cell inoculation. **(D)** Column chart quantifying colony formation after 14 days of co-culture with tomato lycopene-treated cells. ns *p* ≥ 0.05; *p < 0.05; **p < 0.01; ***p < 0.005; ****p < 0.001.

To evaluate lycopene’s potential to inhibit PDAC cell proliferation, this study first investigated the effects of varying lycopene concentrations on two distinct PDAC cell lines (PANC-1 and SW1990) and two normal human pancreatic duct epithelial cell lines (HPNE and HPDE6-C7). The results indicated that after 48 and 72 h of treatment, PDAC cell viability was significantly reduced, whereas HPNE and HPDE6-C7 cells exhibited minimal effects. After 48 h of treatment, the IC₅₀ values for PANC-1, SW1990, HPNE, and HPDE6-C7 cells were 32.16 μM, 63.22 μM, 229.2 μM, and 138.3 μM, respectively. After 72 h of treatment, the IC₅₀ values for these cell lines were 44.82 μM, 76.59 μM, 280.5 μM, and 145.4 μM, respectively (Figure 6A). Based on these results, DMSO vehicle control and 20 and 40 μM lycopene were selected for subsequent functional experiments. Although 20 and 40 μM were below the 48 h IC₅₀ value of SW1990 cells, these sub-IC₅₀ concentrations were selected to reduce the possibility that changes in migration, colony formation, EMT markers, and signaling proteins were simply caused by nonspecific cytotoxicity. The same concentrations were used in PANC-1 and SW1990 cells to allow direct comparison between the two PDAC cell lines and to maintain a safety margin relative to normal pancreatic duct epithelial cells. Nevertheless, these concentrations were used for mechanistic *in vitro* evaluation and should not be interpreted as concentrations that are directly achievable through ordinary dietary lycopene intake in humans. The results demonstrated that lycopene exhibited stronger growth inhibitory effects on PDAC cells compared to normal human pancreatic ductal epithelial cells. Cell growth curve analysis showed that lycopene effectively inhibited the growth of both PDAC cell lines (Figure 6B). Moreover, both 20 μM and 40 μM lycopene significantly suppressed colony formation in PANC-1 and SW1990 cells (*p* < 0.001) (Figures 6C,D).

### 3.7 Lycopene inhibits PDAC cell migration and modulates EMT-associated marker expression

**Figure 7:**
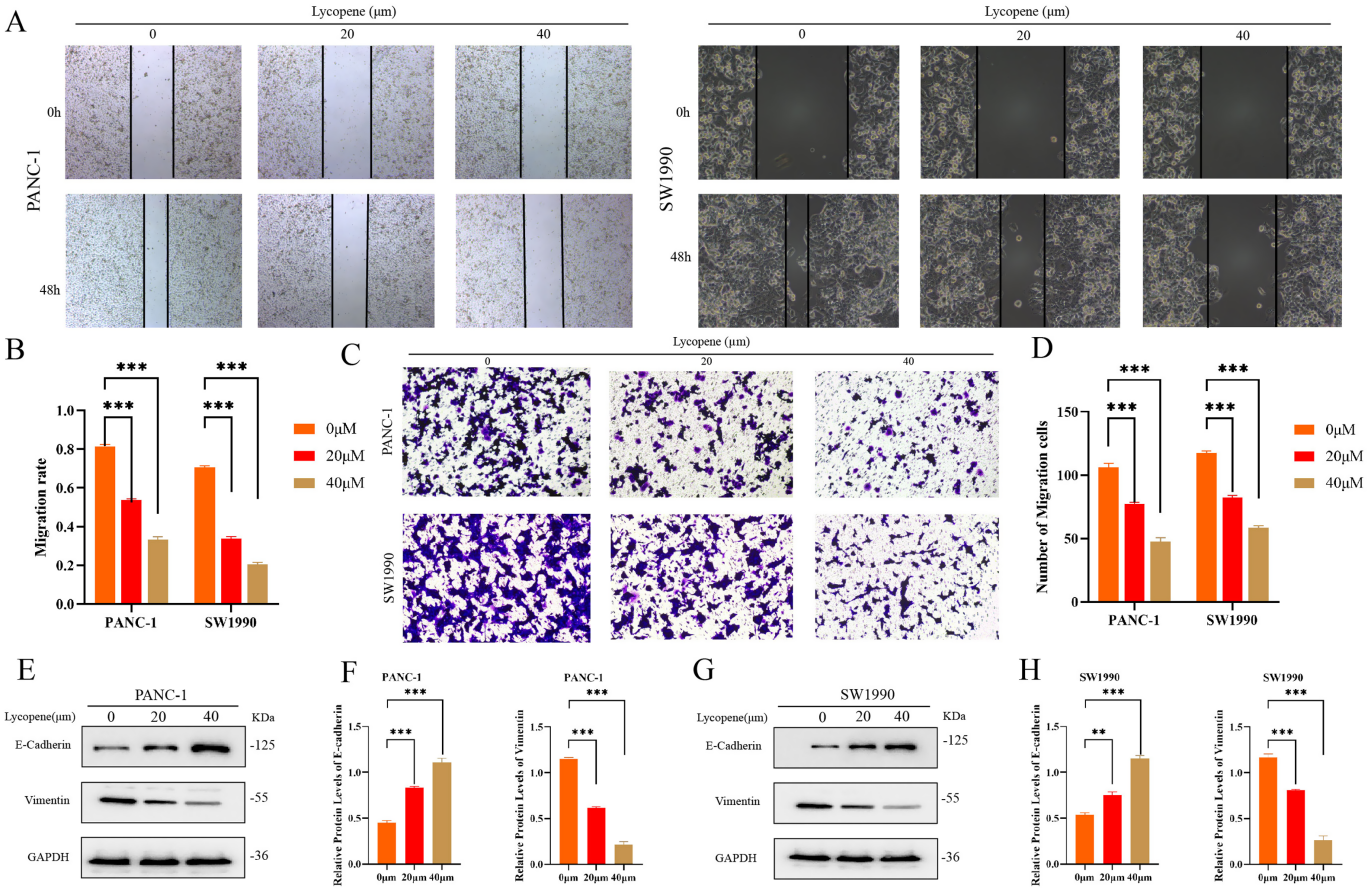
Lycopene inhibits PDAC cell migration. **(A,B)** Wound healing assays were used to calculate migration rates. **(C,D)** Transwell assays assessed the effects of lycopene treatment on PDAC cells. **(E,F)** Western blot analysis of E-cadherin and vimentin in PANC-1 cells. **(G,H)** Western blot analysis of E-cadherin and vimentin in SW1990 cells. ns *p* ≥ 0.05; *p < 0.05; **p < 0.01; ***p < 0.005; ****p < 0.001.

We investigated the potential inhibitory effect of lycopene on PDAC cell migration using the scratch assay and Transwell experiments. As shown in (Figures 7A–D), untreated PANC-1 and SW1990 cells exhibited strong migratory capacity. However, lycopene treatment significantly and concentration-dependently inhibited migration in both cell lines. Furthermore, we explored whether lycopene’s inhibitory effect on PDAC cell migration was associated with changes in EMT-related phenotypic markers. To this end, we assessed the expression levels of EMT marker proteins following lycopene treatment. Western blot analysis showed that lycopene increased the expression of the epithelial marker E-cadherin and decreased the expression of the mesenchymal marker vimentin (Figures 7E–H). Collectively, these findings suggest that lycopene attenuates PDAC cell migration and is associated with partial reversal of EMT-related marker expression. However, because EMT transcription factors were not examined, these results should be interpreted as EMT-associated phenotypic changes rather than direct evidence that lycopene suppresses the EMT transcriptional program.

### 3.8 Lycopene promotes apoptosis-associated cell death in PDAC cells

**Figure 8:**
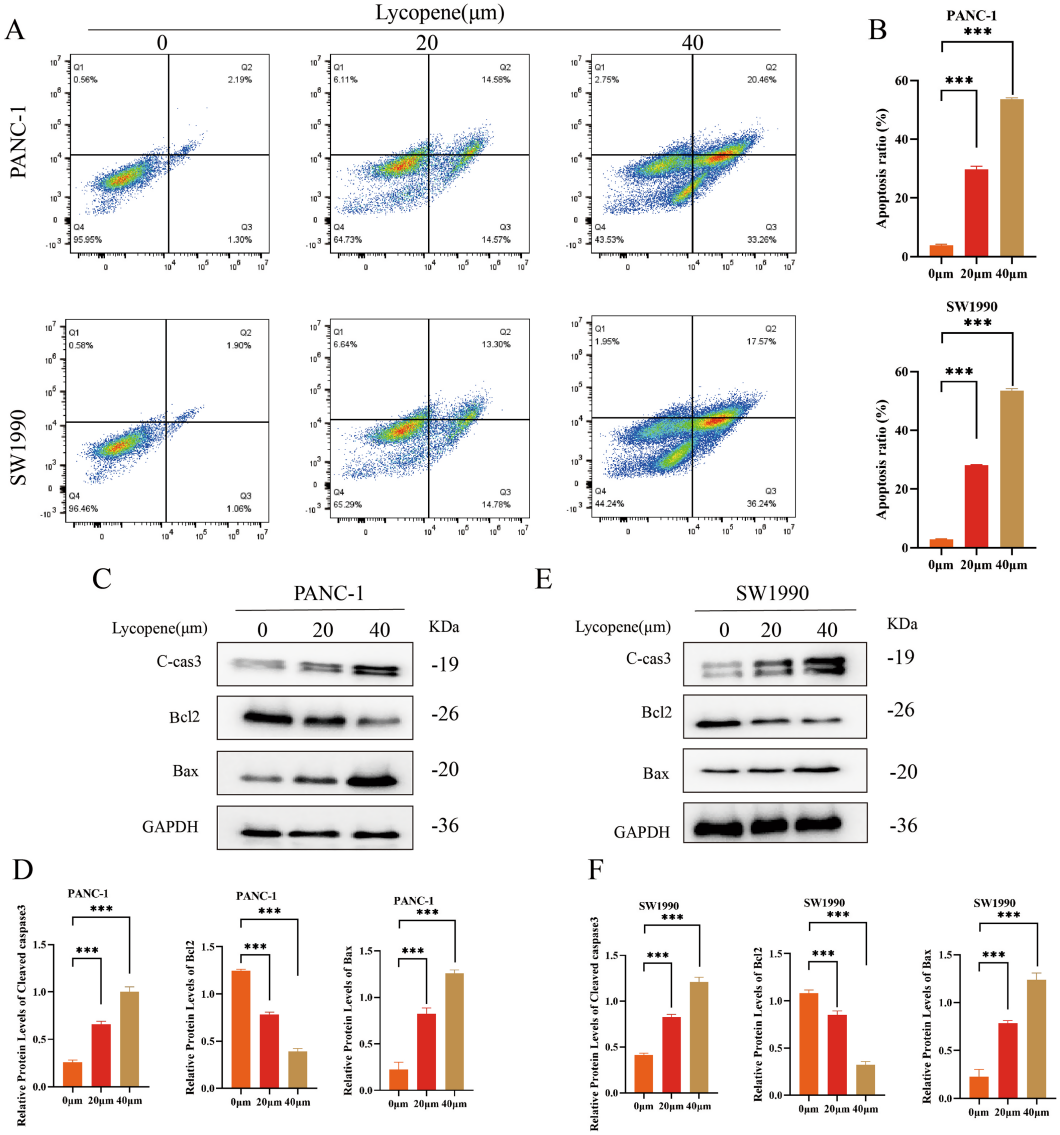
Lycopene-induced apoptosis in PDAC cells and regulation of apoptosis-related protein expression. **(A)** Flow cytometry analysis of lycopene’s effect on apoptosis in PDAC cells. **(B)** Quantitative statistical analysis of apoptosis rate. **(C)** Western blot analysis of apoptosis-related protein expression in PANC-1 cells. **(D)** Quantitative analysis of apoptosis-related protein expression in PANC-1 cells. **(E)** Western blot analysis of apoptosis-related protein expression in SW1990 cells. **(F)** Quantitative analysis of apoptosis-related protein expression in SW1990 cells. ns *p* ≥ 0.05; *p < 0.05; **p < 0.01; ***p < 0.005; ****p < 0.001.

We further investigated whether lycopene could induce apoptosis in PDAC cells. As shown in Figures 8A,B, Annexin V/7-AAD flow cytometry showed that lycopene treatment increased Annexin V-positive cell populations in both PDAC cell lines. Because an increase in 7-AAD-positive cells was also observed, these data were interpreted as apoptosis-associated cell death rather than definitive evidence of apoptosis alone. Additionally, previous network pharmacology and molecular docking analyses suggested that BCL2 may be one of the apoptosis-related hub targets associated with the potential anti-PDAC activity of lycopene. As shown in Figures 8C–F, western blot analysis reveals that lycopene treatment suppresses BCL2 expression while increasing the levels of Bax and cleaved caspase-3 proteins. These findings suggest that lycopene promotes apoptosis-associated cell death in PDAC cells, although the contribution of secondary necrosis or nonspecific cytotoxicity cannot be fully excluded based on the current flow cytometry data alone.

### 3.9 Focused experimental validation of the PI3K/Akt/P53 axis

The network pharmacology analysis identified several hub genes potentially involved in the anti-PDAC effects of lycopene, including AKT1, TP53, BCL2, CASP3, IL6, IL1B, TNF, JUN, STAT3, and NFKB1. These targets are associated with multiple biological processes and signaling pathways, including apoptosis, PI3K/Akt signaling, and inflammation-related pathways. In the present study, we performed focused experimental validation of the PI3K/Akt/P53 axis and apoptosis-related proteins because AKT1, TP53, BCL2, and CASP3 were highly ranked in the PPI network, the PI3K-Akt pathway was strongly enriched in KEGG analysis, and these molecules were closely related to the observed phenotypes of reduced proliferation and apoptosis-associated cell death. In contrast, inflammation-related hub targets, including IL6, IL1B, TNF, JUN, STAT3, and NFKB1, were not experimentally validated in the present study and should therefore be interpreted as computationally predicted candidate targets for future investigation.

**Figure 9:**
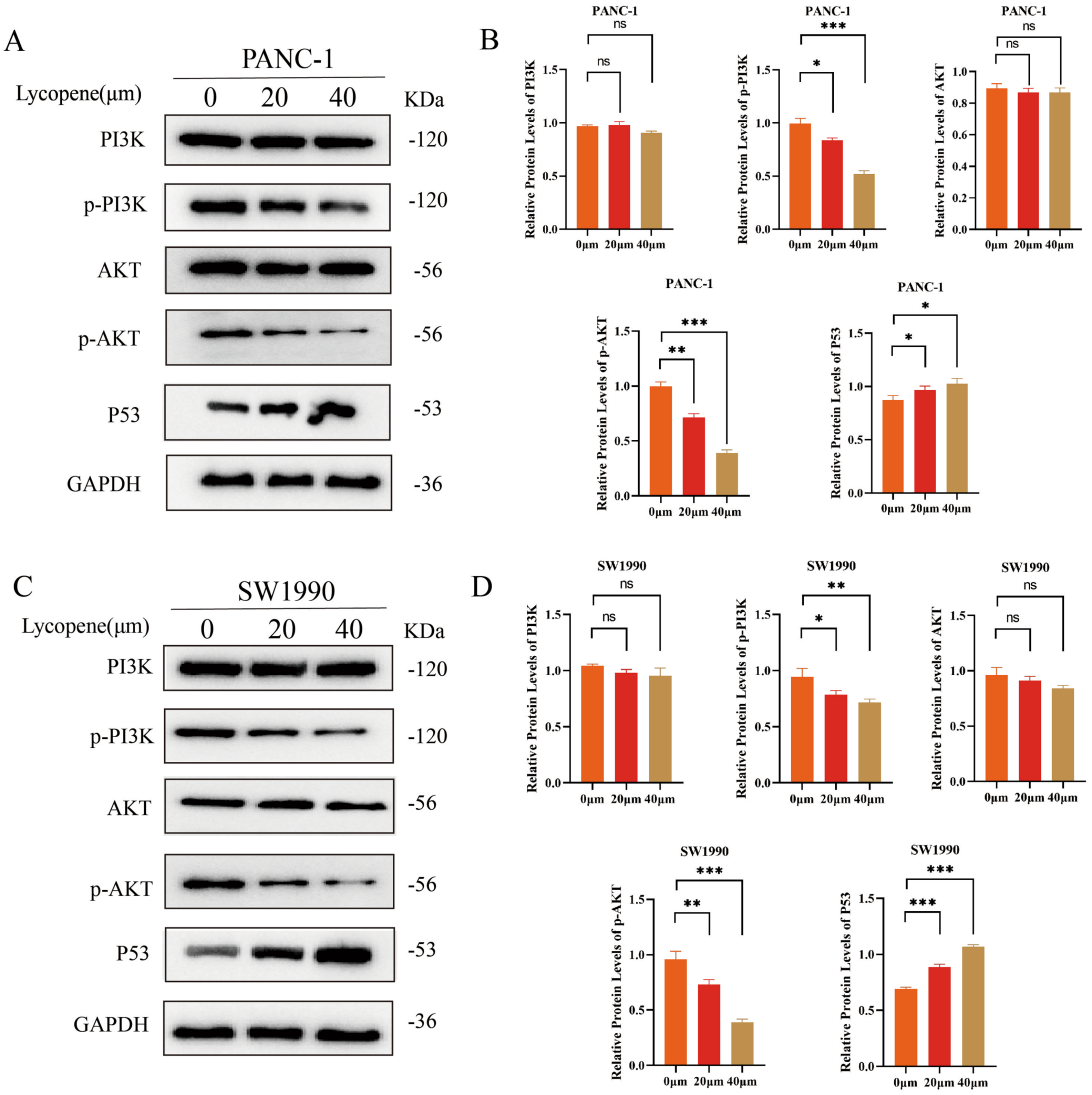
Lycopene inhibits the PI3K/AKT signaling pathway and upregulates p53 expression in pancreatic cancer cells. **(A)** Western blot analysis of PI3K, p-PI3K, AKT, p-AKT, and p53 protein expression in PANC-1 cells treated with 0, 20, or 40 μM lycopene. GAPDH was used as the loading control. **(B)** Quantification of relative protein levels of PI3K, p-PI3K, AKT, p-AKT, and p53 in PANC-1 cells treated with 0, 20, or 40 μM lycopene. **(C)** Western blot analysis of PI3K, p-PI3K, AKT, p-AKT, and p53 protein expression in SW1990 cells treated with 0, 20, or 40 μM lycopene. GAPDH was used as the loading control. **(D)** Quantification of relative protein levels of PI3K, p-PI3K, AKT, p-AKT, and p53 in SW1990 cells treated with 0, 20, or 40 μM lycopene. ns *p* ≥ 0.05; *p < 0.05; **p < 0.01; ***p < 0.005; ****p < 0.001.

Western blot analysis showed that lycopene decreased the expression levels of p-PI3K and p-AKT in both PANC-1 and SW1990 cells, whereas total PI3K and total AKT levels were not significantly changed. Meanwhile, P53 expression was increased after lycopene treatment (Figures 9A–D). Together with the apoptosis-related protein changes described above, these results suggest that lycopene treatment is associated with modulation of apoptosis-related proteins and the PI3K/AKT/P53 signaling axis in PDAC cells. However, because pathway rescue experiments were not performed, the current data support pathway association rather than a confirmed causal PI3K/AKT/P53-dependent mechanism.

## 4 Discussion

PDAC is one of the most aggressive malignancies of the digestive system and remains associated with a poor prognosis, largely because early diagnosis is difficult and most patients are diagnosed at an advanced stage (18). Early diagnosis remains a significant challenge, with most cases being detected at an advanced stage, which complicates treatment significantly (19). In recent years, advances in multidisciplinary treatment approaches have markedly improved overall survival and metastasis-free survival rates for patients with locally resectable tumors (20). However, over 80% of patients are diagnosed at an inoperable, advanced stage, which severely limits the possibility of curative surgical resection (21). The introduction of new chemotherapy regimens combining albumin-bound paclitaxel with gemcitabine and FOLFIRINOX has led to moderate improvements in patient survival (22). Despite these advances in comprehensive PDAC treatment, particularly among borderline resectable patients, the development of both primary and secondary drug resistance continues to limit the effectiveness of existing regimens, resulting in only modest improvements in long-term survival (23). Consequently, there is an urgent clinical need for the development of safe and effective novel natural therapies to address the ongoing treatment challenges in PDAC.

Extensive evidence indicates that lycopene is one of the most potent naturally occurring carotenoids, exhibiting strong antioxidant, anti-inflammatory, lipid metabolism-regulating, and anti-aging properties (24). Further emerging studies highlight its substantial potential for preventing cancer. Current understanding suggests that the anticancer effects of lycopene are mediated through both antioxidant and non-oxidative mechanisms. As a powerful antioxidant, lycopene efficiently quenches singlet oxygen and scavenges various free radicals, thereby reducing oxidative damage to lipids, proteins, and DNA. In addition to its antioxidant properties, lycopene has anti-cancer effects via non-oxidative pathways. These include regulating the phosphorylation of tumor suppressor-associated proteins, inducing cell cycle arrest, modulating metabolic enzymes, and inhibiting insulin-like growth factor-mediated cell proliferation (25, 26). Rowles et al. (27) reported that lycopene intake or circulating lycopene levels are inversely associated with the risk of multiple cancers, including breast, colorectal, lung, and prostate cancers. Compared with other carotenoids, lycopene demonstrates greater efficacy in inhibiting breast cancer cell proliferation and promoting apoptosis. Notably, lycopene uniquely disrupts cytoskeletal organization, selectively arresting cells in specific phases of the cell cycle and ultimately suppressing proliferation. Animal studies by Langner et al. (28) further confirmed that lycopene mitigates the progression of colon and bladder cancer lesions. Despite these advances, research into the impact of lycopene on PDAC remains limited. Therefore, this study aimed to explore the potential anticancer activity of lycopene against PDAC. To clarify the potential mechanisms of lycopene activity, we used network pharmacology and molecular docking to predict candidate targets and pathways, while molecular dynamics simulations were used only as supportive computational analyses for selected representative docking complexes. Enrichment analyses based on key targets, GO functions, and KEGG pathways were performed to identify the principal biological processes and signaling pathways associated with lycopene’s anti-PDAC activity. Furthermore, to confirm lycopene’s anticancer properties and investigate its regulatory influence on important signaling pathways, *in vitro* tests were carried out. In our preliminary analysis, we identified 132 potential targets through which lycopene may exert therapeutic effects against PDAC. Subsequently, a PPI network was constructed, and topological analysis was performed to further screen and identify six core targets closely associated with PDAC progression: TP53, AKT1, TNF, BCL2, IL6, and IL1B.

By controlling cell cycle arrest and death, TP53, commonly known as the “guardian of the genome,” performs a vital tumor-suppressive function in preserving genomic integrity. In PDAC, the mutation rate of TP53 is remarkably high, reaching approximately 70–80% (29). Upon mutation, p53 not only loses its canonical tumor-suppressive function but may also acquire oncogenic gain-of-function properties. Emerging evidence indicates that mutant p53 can interact with YAP1 to promote tumor stemness and metastasis, constituting a key mechanism underlying the malignant progression of PDAC (30). AKT, also known as protein kinase B, is a pivotal cytoplasmic serine/threonine kinase that regulates apoptosis and cell survival signaling. In mammals, AKT comprises three isoforms-AKT1, AKT2, and AKT3-with AKT1 being ubiquitously expressed across most tissues (31). In PDAC, AKT1 frequently exhibits elevated expression and aberrant activation. Notably, levels of phosphorylated AKT1 (p-AKT1) in the cytoplasm and nucleus of PDAC cells are significantly higher than those in normal ductal epithelium, suggesting that AKT1 activation is closely linked to the malignant characteristics of PDAC. The overexpression and constitutive activation of AKT1 promote PDAC cell proliferation, suppress apoptosis, and enhance tumor cell survival, thereby playing a central oncogenic role in tumor initiation and progression. Consequently, targeting AKT1 represents a promising therapeutic strategy for PDAC (32). Inhibiting AKT1 expression or activity could be an effective way to delay disease progression and improve clinical outcomes (33). TNF is a multifunctional cytokine that exerts both pro-inflammatory and immunomodulatory effects in inflammatory diseases and cancer. Primarily secreted by cytotoxic lymphocytes and other immune cells, TNF can contribute to pathological processes in various malignancies and autoimmune disorders. Activation of the TNF signaling pathway has been shown to promote PDAC progression (34). Accordingly, recent studies have investigated the expression patterns and clustering characteristics of TNF family members in PDAC, identifying them as potential key biomarkers for prognostic evaluation. Such stratification strategies may facilitate the prediction of patient prognosis and immune landscape in PDAC (35). BCL2, a core apoptosis-regulating gene localized to the mitochondrial outer membrane, contributes to tumor progression in PDAC by inhibiting mitochondrial apoptotic pathways and sustaining cell survival. Its overexpression markedly decreases the sensitivity of PDAC cells to chemotherapeutic agents, thereby mediating therapeutic resistance (36). As multifunctional cytokines and key inflammatory mediators, IL-6 and IL-1β are involved in a variety of cellular processes, including proliferation, differentiation, and apoptosis. Emerging evidence indicates that IL-1β serves as a critical driver of stromal remodeling and connective tissue hyperplasia in PDAC models (37). Collectively, these six core targets play a pivotal role in the initiation, progression, and prognosis of PDAC, and thus represent potential molecular mediators of lycopene’s anti-PDAC effects. Molecular docking suggested that lycopene may interact with several representative hub targets, and molecular dynamics simulations further provided supportive evidence for the conformational stability of selected high-ranking complexes. However, these computational analyses do not prove direct biochemical binding between lycopene and these targets. Nevertheless, the precise mechanisms by which these key targets contribute to PDAC pathogenesis and the detailed molecular basis of their interactions with lycopene warrant further in-depth investigation.

KEGG enrichment analysis demonstrated that the six core targets were predominantly enriched in the PI3K/Akt signaling pathway, apoptosis pathway, and Toll-like receptor signaling pathway, all of which are closely implicated in tumorigenesis and cancer progression. In the present study, we primarily focused our experimental investigations on the PI3K/Akt pathway. The PI3K/Akt signaling cascade plays a central role in regulating fundamental cellular processes, including proliferation, growth, survival, and angiogenesis (38). In the context of cancer, the aberrant activation or dysregulation of the PI3K/Akt pathway is widely recognized as a critical driver of tumor development and has therefore emerged as an important therapeutic target (39). Previous research by Tang et al. showed that combined supplementation with lycopene and eicosapentaenoic acid inhibited the proliferation of HT-29 human colon cancer cells, which was associated with modulation of PI3K/Akt/mTOR-related signaling (40). Similarly, Wang et al. reported that lycopene inhibits EMT via the PI3K/AKT signaling pathway and promotes apoptosis in oral cancer cells (41). Consistent with previous findings, the present study showed that lycopene treatment was accompanied by decreased p-PI3K and p-AKT expression and increased P53 expression in PDAC cells, suggesting that the PI3K/Akt/P53 signaling axis may be involved in the anti-PDAC effects of lycopene. To validate the results of the network pharmacology analysis further, we conducted *in vitro* experiments. According to data from Western blotting, CCK-8 tests, colony formation assays, wound healing assays, and Transwell migration experiments, lycopene dramatically reduces the proliferative and migration potential of PDAC cells. One crucial stage in the development of tumors is EMT. During this process, epithelial cancer cells undergo phenotypic reprogramming to acquire mesenchymal characteristics, resulting in enhanced migratory and invasive abilities. EMT plays a pivotal role in tumor initiation, malignant progression, and metastasis. Moreover, cancer cells undergoing EMT frequently acquire additional features, including stem cell-like properties, immune evasion capacity, and resistance to therapeutic agents (42, 43). These phenotypic alterations ultimately contribute to treatment failure and tumor recurrence. Previous studies have established that EMT plays a critical role in PDAC progression. Consistent with this, our findings showed that lycopene suppressed PDAC cell migration and was accompanied by increased E-cadherin expression and decreased vimentin expression, suggesting that lycopene may modulate EMT-associated phenotypic changes in PDAC cells. Apoptosis represents a tightly regulated form of programmed cell death that ensures the efficient and orderly elimination of damaged or dysfunctional cells. Enrichment analysis in the present study revealed significant involvement of the apoptosis pathway among the identified core target genes. Accordingly, apoptosis was assessed using Annexin V-FITC/PI dual staining followed by flow cytometric analysis. The findings showed that lycopene treatment increased Annexin V-positive cell populations in both PDAC cell lines. However, because 7-AAD-positive populations were also increased, these results should be interpreted as apoptosis-associated cell death, and the possibility of secondary necrosis or nonspecific cytotoxicity cannot be fully excluded. Earlier research has shown that Bax promotes the permeabilization of the mitochondrial outer membrane and subsequent mitochondrial dysfunction during apoptosis by activating the intrinsic apoptotic pathway. Concurrently, downregulation of BCL2 enhances mitochondrial destabilization and facilitates the release of reactive oxygen species and lipid peroxides, thereby promoting tumor cell apoptosis (44, 45). Cleaved caspase-3, a critical effector protease in the downstream caspase cascade, serves as a central executor of the apoptotic process (46). In conclusion, our results suggest that lycopene promotes apoptosis-related molecular changes in PDAC cells, as reflected by increased cleaved caspase-3 and Bax expression and decreased BCL2 expression. Nevertheless, additional assays are needed to distinguish apoptosis from secondary necrosis more rigorously. Although the PPI analysis identified several hub genes, including inflammatory mediators such as IL6, IL1B, and TNF, the present experimental validation focused on the PI3K/AKT/P53 axis and apoptosis-related proteins because these targets were highly ranked in the network analysis, strongly enriched in KEGG pathway analysis, and closely associated with the observed phenotypes of inhibited proliferation and enhanced apoptosis. Future studies will further validate the roles of inflammation-related hub targets, such as IL6, IL1B, TNF, JUN, STAT3, and NFKB1, in lycopene-mediated anti-PDAC effects.

Although the PPI analysis identified multiple hub genes, including inflammation-related mediators and transcriptional regulators such as IL6, IL1B, TNF, JUN, STAT3, and NFKB1, the present experimental validation focused on the PI3K/Akt/P53 axis and apoptosis-related proteins. This focused validation strategy was adopted because the PI3K-Akt pathway was strongly enriched in KEGG analysis and was closely associated with the observed phenotypes of inhibited proliferation and apoptosis-associated cell death. Therefore, the current experimental data support the involvement of apoptosis-related proteins and PI3K/Akt/P53 signaling, but they do not establish the functional contribution of the inflammation-related hub targets. The inflammation-related targets identified in the network analysis should be regarded as candidate mediators and hypothesis-generating findings. Future studies should validate IL6, IL1B, TNF, JUN, STAT3, and NFKB1 using qRT-PCR, ELISA, Western blotting, and target-specific rescue or knockdown experiments.

An important translational issue is that the *in vitro* concentrations of lycopene used in the present study may not be directly achievable through ordinary dietary intake. Although 20 and 40 μM lycopene were selected as sub-IC₅₀ concentrations for functional assays, especially for SW1990 cells, these concentrations are higher than the typical plasma concentrations reported in humans after dietary lycopene intake. Therefore, the present findings should be interpreted as mechanistic *in vitro* evidence rather than direct evidence of clinically achievable efficacy through dietary lycopene alone. The clinical translation of lycopene is limited by its poor aqueous solubility, lipophilic nature, food matrix-dependent absorption, isomerization status, and relatively low systemic bioavailability. Nevertheless, formulation strategies such as lipid-based delivery systems, nanoemulsions, nanostructured lipid carriers, self-emulsifying delivery systems, and cis-isomer-enriched or food-matrix-optimized formulations may improve lycopene solubility, intestinal absorption, lymphatic transport, and systemic exposure. Whether these approaches can achieve therapeutically relevant concentrations in pancreatic tissue or PDAC lesions remains unclear and should be evaluated in future pharmacokinetic, biodistribution, animal efficacy, and clinical studies. In summary, the present study suggests that lycopene may inhibit PDAC cell proliferation and migration and promote apoptosis-associated cell death. These effects were accompanied by changes in apoptosis-related proteins and PI3K/Akt/P53 signaling components. Meanwhile, network pharmacology and molecular docking identified additional candidate hub targets, including inflammation-related molecules such as IL6, IL1B, TNF, JUN, STAT3, and NFKB1, which require further experimental validation. These findings suggest that lycopene may suppress PDAC cell proliferation and migration and modulate EMT-associated marker expression, while promoting apoptosis-associated cell death. This provides preliminary insight into its molecular mechanisms against PDAC. Nevertheless, several limitations should be acknowledged. First, this study was mainly based on *in vitro* cellular experiments and computational analyses, without validation in animal models, clinical specimens, or clinical settings. Key pharmacological issues, including the optimal dosage, formulation, bioavailability, tissue distribution, and *in vivo* metabolic behavior of lycopene, were not fully investigated. In particular, the concentrations used in the *in vitro* experiments were higher than typical plasma concentrations achievable after ordinary dietary intake in humans. Therefore, the current results should be interpreted as preliminary mechanistic evidence rather than direct proof of clinical efficacy. Future studies should determine whether optimized lycopene formulations, such as lipid-based formulations, nanoemulsions, nanostructured lipid carriers, or self-emulsifying delivery systems, can improve systemic exposure and achieve biologically effective concentrations in pancreatic tissue or tumor lesions. Second, although DMSO vehicle controls were used and the final DMSO concentration was kept constant at 0.1% v/v in all groups, the potential influence of DMSO on PI3K/Akt signaling cannot be completely excluded. The concentrations of 20 and 40 μM lycopene were selected as sub-IC₅₀ doses, especially for SW1990 cells, to reduce nonspecific cytotoxicity; however, dose-dependent pathway regulation requires further validation. In addition, EMT transcription factors, such as Snail, Slug, Twist, ZEB1, and ZEB2, were not examined in the present study. Therefore, the observed increase in E-cadherin and decrease in vimentin should be interpreted as changes in EMT-associated phenotypic markers rather than direct evidence that lycopene regulates EMT transcriptional programs. Future studies should evaluate EMT transcription factors and perform functional perturbation experiments to clarify whether lycopene directly modulates EMT regulatory networks in PDAC cells. Third, Annexin V/7-AAD flow cytometry suggested apoptosis-associated cell death, but the increase in 7-AAD-positive cells indicates that secondary necrosis or nonspecific cytotoxicity may also contribute to the observed cell death. Fourth, rescue experiments were not performed; therefore, the observed changes in p-PI3K, p-AKT, and P53 should be interpreted as pathway-associated effects rather than definitive evidence of a causal PI3K/Akt/P53-dependent mechanism. Fifth, although IL6, IL1B, TNF, JUN, STAT3, and NFKB1 were identified as important inflammation-related hub targets in the network analysis, they were not experimentally validated in the present study. Therefore, conclusions regarding inflammation-related mechanisms remain computational and hypothesis-generating. In addition, the molecular dynamics simulations were performed only for BCL2-lycopene and TP53-lycopene, without AKT1-lycopene simulation, apo-protein controls, or ligand-specific stability analyses. Thus, future studies should include *in vivo* validation, clinical specimen analysis, pathway-rescue experiments, AKT1-related computational validation, and biochemical binding assays such as CETSA, DARTS, SPR, or ITC to further confirm the anti-PDAC mechanism of lycopene.

## 5 Conclusion

This study employed network pharmacology, molecular docking, representative molecular dynamics simulations, and focused *in vitro* validation to investigate the potential anti-PDAC activity of lycopene. The results suggest that lycopene may inhibit PDAC cell proliferation and migration and promote apoptosis-associated cell death, accompanied by changes in apoptosis-related proteins and PI3K/Akt/P53 signaling components. Inflammation-related hub targets, including IL6, IL1B, TNF, JUN, STAT3, and NFKB1, were identified as additional computational candidates and warrant further experimental validation. Importantly, because the effective *in vitro* concentrations of lycopene exceed typical plasma concentrations achievable through ordinary dietary intake, further formulation optimization, pharmacokinetic evaluation, and *in vivo* validation are required before its translational potential in PDAC can be established.

## Data Availability

The original contributions presented in the study are included in the article/Supplementary material, further inquiries can be directed to the corresponding author/s.

## Footnotes

1
https://pubchem.ncbi.nlm.nih.gov/

2
https://www.Ctdbase.org/

3
https://www.ebi.ac.uk/chembl/

4
http://herb.ac.cn/

5
https://portal.gdc.cancer.gov/

6
https://www.ncbi.nlm.nih.gov/geo/

7
https://www.genecards.org/

8
https://string-db.org/
