# Supplementary material for: Integrating network pharmacology, molecular docking, and experimental validation to investigate the therapeutic effects and potential mechanisms of lycopene against pancreatic ductal adenocarcinoma
Source: Front Nutr. 2026 Jul 14;13:1815515. doi: 10.3389/fnut.2026.1815515 (PMC13410675; doi:10.3389/fnut.2026.1815515)
Supplement: Supplementary file 1 [file Table_1.DOCX]

**Supplementary Table S1. Summary of PDAC-specific studies identified in the secondary search on lycopene and PDAC**

| **Num.** | **First author/year** | **Study type** | **Disease/model** | **Main focus** | **Key findings** | **Relevance to the present study** |
| --- | --- | --- | --- | --- | --- | --- |
| 1 | Dalbayrak et al., 2026 | Original research / in vitro study | Human pancreatic cancer cell lines PANC-1 and MIA PaCa-2, including wild-type and miR-21 knockout cells | Chemopreventive effects of lycopene in pancreatic cancer cells and the role of miR-21 | Lycopene treatment at 50 μM reduced cell viability, colony formation, migration, spheroid integrity, and intracellular ROS levels. The inhibitory effects were more pronounced in miR-21-deficient cells. | Provides direct experimental evidence that lycopene suppresses malignant phenotypes of pancreatic cancer cells and regulates ROS-related biological responses, supporting the rationale for studying lycopene in PDAC. |
|  |  |  |  |  |  |  |
| 2 | Luo et al., 2025 | Systematic review and meta-analysis of Mendelian randomization studies | Pancreatic cancer / PDAC risk factors | Genetically proxied risk and protective factors for pancreatic cancer | Lycopene intake was identified as a potential protective factor for pancreatic cancer, with an OR of 0.87 and 95% CI of 0.77-0.99 in the IVW meta-analysis. The authors noted that further validation is still needed. | Provides population-level genetic epidemiological evidence suggesting a possible inverse association between lycopene intake and pancreatic cancer risk. |
|  |  |  |  |  |  |  |
| 3 | Maaz et al., 2025 | Review | Multiple cancer types, including pancreatic cancer and pancreatic cell models | Molecular mechanisms of lycopene in cancer therapy | The review summarized that lycopene may reduce ROS, inhibit NF-κB-related signaling, suppress inflammatory mediators such as IL-6 and TNF-α, and regulate cancer-related pathways including PI3K/Akt/mTOR. In the pancreatic cancer section, lycopene was reported to reduce intracellular and mitochondrial ROS and inhibit NF-κB target genes in PANC-1 cells. | Provides mechanistic background linking lycopene with oxidative stress, inflammatory signaling, and pancreatic cancer-related molecular pathways, supporting the biological plausibility of lycopene as an anti-PDAC compound. |
|  |  |  |  |  |  |  |
| 4 | Zhang et al., 2022 | Mendelian randomization study | Digestive system cancers, including pancreatic cancer | Causal associations between diet-derived circulating antioxidants and digestive system cancers | This study evaluated several diet-derived antioxidants, including lycopene, in relation to digestive system cancers. Overall, genetically determined antioxidants were not significantly associated with digestive system cancers after multiple testing correction. Suggestive protective evidence was observed for retinol, rather than lycopene, in pancreatic cancer. | Provides genetic epidemiological evidence regarding antioxidants and pancreatic cancer risk. Although it does not directly support a causal protective role of lycopene, it highlights the need for mechanistic and experimental validation of lycopene in PDAC. |
| 5 | Jeong et al., 2019 | Original research / in vitro study | Human pancreatic cancer PANC-1 cells | Lycopene, ROS-mediated NF-κB signaling, and apoptosis | Lycopene decreased intracellular and mitochondrial ROS levels, reduced mitochondrial function, suppressed NF-κB activity and NF-κB-dependent survival genes, including cIAP1, cIAP2, and survivin. Lycopene also reduced cell viability and increased active caspase-3 and the Bax/Bcl-2 ratio. | Provides direct mechanistic evidence that lycopene can inhibit ROS/NF-κB-mediated survival signaling and induce apoptosis in pancreatic cancer cells, supporting the mechanistic basis of the present study. |
|  |  |  |  |  |  |  |
| 6 | Huang et al., 2016 | Systematic review and meta-analysis of epidemiological studies | Human pancreatic cancer | Association between vitamin A, retinol, carotenoid intake, and pancreatic cancer risk | Dietary lycopene intake was inversely associated with pancreatic cancer risk, with a pooled OR of 0.84, 95% CI 0.73-0.97, *P* = 0.020. The inverse association was more evident in case-control studies. | Provides population-level epidemiological evidence suggesting that lycopene intake may be inversely associated with pancreatic cancer risk, supporting the disease relevance of lycopene. |
|  |  |  |  |  |  |  |
| 7 | Tarasiuk and Fichna, 2019 | Review | Acute pancreatitis models | Therapeutic value of phytochemicals, including lycopene, in acute pancreatitis | The review summarized that lycopene pretreatment at 50 mg/kg in a cerulein-induced acute pancreatitis rat model protected pancreatic tissue from oxidative damage by inhibiting neutrophil infiltration and lipid peroxidation. | Provides pancreatic tissue-related evidence supporting the antioxidant and anti-inflammatory relevance of lycopene in pancreatic injury, although it is not a direct PDAC study. |
|  |  |  |  |  |  |  |
| 8 | Chen et al., 2016 | Meta-analysis | Human pancreatic cancer | Association between antioxidant intake and pancreatic cancer risk | This meta-analysis included 18 studies. Lycopene intake was marginally associated with reduced pancreatic cancer risk, with a pooled OR of 0.85 and 95% CI of 0.73-1.00. | Provides epidemiological evidence suggesting a possible inverse association between lycopene intake and pancreatic cancer risk, although the association was borderline. |
|  |  |  |  |  |  |  |
| 9 | Han et al., 2013 | Prospective cohort study | Human pancreatic adenocarcinoma | Dietary and supplemental antioxidant intake and pancreatic cancer risk | The study included 77,446 participants and 184 pancreatic adenocarcinoma cases. Seven antioxidants, including lycopene, were evaluated. A significant inverse association was observed for dietary selenium, whereas lycopene was not identified as a significant protective factor. | Provides prospective cohort evidence indicating that antioxidant-related pancreatic cancer prevention remains inconsistent; useful as a neutral/negative reference for lycopene. |
| 10 | Jeurnink et al., 2014 | Nested case-control study within EPIC | Human exocrine pancreatic cancer | Plasma carotenoids, vitamin C, retinol, tocopherols, and pancreatic cancer risk | The study included 446 pancreatic cancer cases and 446 matched controls. Higher plasma β-carotene, zeaxanthin, and α-tocopherol were inversely associated with pancreatic cancer risk, whereas plasma lycopene was not significantly associated with pancreatic cancer risk. | Provides biomarker-based epidemiological evidence. Although lycopene itself was not significantly associated with risk, the study supports the broader relevance of carotenoids and oxidative stress-related nutrients in pancreatic cancer etiology. |
|  |  |  |  |  |  |  |
| 11 | Zhang et al., 2011 | Population-based case-control study | Human pancreatic cancer | Antioxidant defense genes, DNA repair genes, dietary antioxidants, and pancreatic cancer risk | This study included 189 pancreatic cancer cases and 486 controls. Variants in oxidative stress-related genes were associated with pancreatic cancer risk. The SOD2 variant allele was associated with reduced risk, whereas the hOGG1 variant allele was associated with increased risk. The protective effect of the SOD2 variant was more pronounced among individuals with low dietary intake of several antioxidants, including lycopene. | Provides evidence that oxidative stress defense and oxidative DNA damage repair are relevant to pancreatic cancer risk. Although it does not directly test lycopene as an intervention, it supports the biological relevance of antioxidant-related mechanisms and lycopene intake in pancreatic cancer etiology. |
|  |  |  |  |  |  |  |
| 12 | Nkondjock et al., 2005 | Population-based case-control study | Human pancreatic cancer | Dietary carotenoid intake and pancreatic cancer risk | This study included 462 histologically confirmed pancreatic cancer cases and 4,721 population-based controls from eight Canadian provinces. Lycopene intake, mainly from tomatoes, was associated with a 31% reduction in pancreatic cancer risk among men, with OR = 0.69, 95% CI 0.46-0.96. The authors suggested that diets rich in tomatoes and tomato-based products may help reduce pancreatic cancer risk. | Provides direct epidemiological evidence linking higher dietary lycopene intake with reduced pancreatic cancer risk, supporting the disease-specific rationale for investigating lycopene in PDAC. |
| 13 | Burney et al., 1989 | Nested case-control study | Human pancreatic cancer | Prediagnostic serum micronutrients and subsequent pancreatic cancer risk | This study analyzed stored prediagnostic serum samples from 22 pancreatic cancer cases and 44 matched controls. Serum lycopene and selenium levels were lower in cases than in controls. In matched analysis, low serum lycopene was associated with increased pancreatic cancer risk, with OR = 6.4 and 95% CI 1.73-24.2. | Provides biomarker-based prospective evidence that lower prediagnostic serum lycopene levels may be associated with subsequent pancreatic cancer risk, supporting the disease-specific relevance of lycopene. |
